# Incidence and outcome for patients with occult lymph node involvement in T1 and T2 oral squamous cell carcinoma: a prospective study

**DOI:** 10.1186/1471-2407-14-346

**Published:** 2014-05-20

**Authors:** Thomas Mücke, David A Mitchell, Stefan Wagenpfeil, Lucas M Ritschl, Klaus-Dietrich Wolff, Anastasios Kanatas

**Affiliations:** 1 Department of Oral and Maxillofacial Surgery, Technische Universität München, Klinikum Rechts der Isar, Ismaninger Str. 22, 81675 Munich, Germany; 2 Department of Oral and Maxillofacial Surgery, Oral and Facial Specialties Department, Mid Yorkshire Hospitals NHS Trust, Aberford Road, WF1 4DG West Yorks, UK; 3 Institute for Medical Biometry, Epidemiology und Medical Informatics (IMBEI), Universitätsklinikum des Saarlandes, Homburg/Saar, Germany

**Keywords:** Oral cancer, Outcome, Lymph node metastasis, Neck dissection

## Abstract

**Background:**

The evidence base to inform the decision making process in patients with early stage oral cancer and a clinical and radiological N0 neck remains insufficient to answer the question when it is safe to “watch and wait” and when to proceed with a selective neck dissection.

**Methods:**

A total of 327 consecutive cases of histopathologically staged T_1–2_, N_0–1_ and M_0_, but clinically N_0,_ squamous cell carcinoma of the tongue were prospectively analysed. Univariate and multivariate analyses were used for statistical analysis and are represented as Kaplan-Meier analyses or Cox proportional hazard regression analysis.

**Results:**

In 61 patients (18.65%) lymph node involvement was found in the histopathological processing. The mean survival of all patients was 73.3 ± 48.6 months. The 2-year and 5-year overall survival rates of all patients were 87.5% and 68.4%, respectively. The 2-year and 5-year survival rates for stage N_0_ were 89.1% and 70.7% compared to 83.3% and 62.9% in N_1_ situations. The 2-year and 5-year survival rates for stage T_1_ were 87.9% and 73.6% compared to 87.2% and 65.3% in stage T_2_, respectively_._ The time to recurrence in stage N_0_ was 35.1 ± 30.5 months compared to 25.63 ± 24.6 months in cases with N_1_ disease. Stage T_1_ was associated with a time to recurrence of 38.1 ± 33.9 months compared with 27.2 ± 22.7 months in patients classified T_2_.

Variables found to be strongly associated with survival in the univariate analysis included older age, higher tumour and N stage, and grading. Age, tumour stage (p = 0.011, 95% CI, 1.09 to 2.0), nodal stage (p = 0.038, 95% CI, 1.02 to 2.07), and recurrence were independently and significantly associated with survival in the multivariate analysis.

**Conclusions:**

This confirms a high overall disease free survival for patients with T1 and N0 treated with single modality surgery and in common with the literature confirms the poor impact on prognosis of the N positive neck.

## Background

The incidence of pathologically positive lymph nodes in the clinically and radiologically negative neck (N0) in T_1_ and T_2_ squamous cell carcinomas (SCC) of the oral cavity remains controversial. A prospective randomised trial is attempting to answer the question of when it is in the best interests of the patient to carry out elective neck dissection (SEND). Several single centre audits have demonstrated an incidence of around 30% positive cervical nodes in clinically and radiologically undetected cases with T2 tongue and floor of mouth squamous cancer. Belief that only thin T2 and T1 SCC should not undergo neck dissection (patient factors permitting) is so strong that recruitment to the SEND study has been slow. Surgery with or without radiation and chemotherapy is the established curative treatment of SCC [1]. Treatment of locally advanced SCC within an isolated organ of the oral cavity is recognised as requiring multi-modal treatment approaches including surgery and radiotherapy with or without chemotherapy [2]. Although ablative surgery with or without reconstruction is an established therapy for small tumours staged at T_1_ and T_2_ created controversies exists around the role of neck dissection. Controversy also exists with radiation therapy which is a single use treatment with lifelong post treatment morbidity [3]. The management decision around small stage T1 and T2 SCC particularly thin tumours centers around either a wait-and-see policy or a selective neck dissection of the ipsilateral lymph nodes of level I-IV,which logically should be bilateral in midline lesions [4,5].

The purpose of the present study was to investigate the oncologic results and role of primary surgical treatment for clinically early-stage SCC. The incidence of lymph node involvement and its role in overall survival was further investigated.

## Methods

### Eligibility

Any patient who had histologically confirmed invasive SCC of the tongue histopathologically staged T_1–2_, N_0–1_ and M_0_ was eligible. Exclusion criteria included previous malignancy at the oral cavity or positive resection margins.

### Ethics and consent

Every patient gave an informed consent to participate in clinical studies regarding the analyses of survival and outcome of their treatments. Due to the informed consent already given by patients to participate in studies and willing to perform the required medical care an exemption from requiring ethics approval was granted by the Ethical Committee of the University of Munich.

### Staging

All patients underwent incisional biopsy, computed tomography (CT) or magnetic resonance imaging (MRI), skeletal scintigraphic surveys, sonography, and thoracic x-ray. Lymph nodes of more than 1 cm with a rounded configuration were regarded as probably involved by imaging criteria. In addition, clinical assessment was controlled by CT and/or MRI scans as well as sonography examination. All diagnostics were performed by an expert radiologist and individual cTNM was confirmed by an interdisciplinary tumour board including specialists from radiology, oral and maxillofacial surgery, ear nose and throat surgeons, oncology, radiation therapy, and nuclear medicine. Postoperative histopathological assessment by the pathologists was used as the diagnostic gold standard, retrospectively.

### Surgery

As part of the staging process all patients underwent histological confirmation of the diagnosis. The extent of neck dissection was performed uni- or bilaterally in level I-III of the neck, depended on the location of metastases intraoperatively [1]. The tumour resection was performed surgically according to current guidelines and as described before followed by microvascular reconstruction [6].

### Follow-up

After surgery, patients were assessed every 3 months for the first 2 years, every 6 months for another 2 years, and every year thereafter. Investigations to detect recurrence were done by clinical inspection and yearly CT routinely.

### Data analysis

Data of the study were prospectively collected in one single department and analysed. Overall survival in months and 5-year overall survival were used as dependent variables. Associations of possible predictor variables with the dependent variable, survival, were determined using the Kaplan-Meier estimator, univariate log-rank test, and Cox proportional hazards regression models. Multiple Cox proportional hazards regression models were conducted to explore the relationship between neck involvement and survival, as were variables shown in the literature to be associated with survival.

Measurements of tumour related differences were compared using the non-parametric Fisher-test, as appropriate. The Kaplan-Meier method was used to plot survival curves for each putative binary prognostic factor. P-values are two-sided and subject to a global significance level of 0.05. The data were analysed with the “Statistical Package for the Social Sciences” (SPSS for Windows, release 21, 2013, SPSS Inc, Chicago, IL, USA).

## Results

### General characteristics

From 1992 to 2008, of the 327 patients matching the criteria were treated. Among these, 266 patients did not show lymph node involvement histopathologically and in 61 patients (18.65%) lymph node involvement was found. The median follow-up was 66 months (range 1–192). Patient demographic, clinical, and tumour characteristics are summarized in Table 1.

**Table 1:** Demographic, Clinical, and Cancer Characteristics of all patients, with and without lymph node involvement

	**All patients**		**Without lymph node involvement**		**With lymph node involvement**		**p-value**
**Characteristic**	**No.**	**%**	**No.**	**%**	**No.**	**%**	
Mean age (years)	60.6		60.25		62.28		
SD	12.45		12.45		12.39		
Range	30-91		30-91		30-91		
Sex							
Woman	105	32.1	82	78.1	23	21.9	0.215
Men	222	67.9	184	82.9	38	17.1	
Performance status							
Dead	129	39.4	98	76.0	31	24.0	0.074
Alive	198	60.6	168	84.8	30	15.2	
Tumor stage							
1	193	59.0	175	90.6	18	9.3	< 0.001*
2	134	41.0	91	67.9	43	32.1	
N stage							
0	266	81.3	266	100			
1	61	18.7			61	100	
Tumor grade							
1	52	15.9	48	92.3	4	7.7	
2	236	72.2	198	83.9	38	16.1	< 0.001*
3	39	11.9	20	51.3	19	48.7	
Recurrence							
0	257	78.6	214	83.3	43	16.7	0.083
1	70	21.4	52	74.3	18	25.7	

Abbreviations: * p < 0.05.

### Outcome

The mean survival of all patients was 73.3 ± 48.6 months. The 2-year overall survival rate of all patients was 87.5%. 5-year overall survival rate of all patients was 68.4%. Among the patients with tumour stages classified as T_1_ the overall survival rates were 87.9% and 73.6% at 2 and 5 years, respectively. The overall survivals in T_2_ tumour stages were 87.2% and 65.3% at 2 and 5 years, respectively. In patients without lymph node involvement the 2-year overall survival rate was 89.1% compared to 83.3% of patient with stage N_1_. The corresponding 5-year overall survival rates were 70.7% for patients without and 62.9% for patients with lymph node involvement at stage N_1_. At the time of analysis, 198 (60.6%) patients were alive and 129 (39.4%) had died. Subdividing patients who were classified as tumour stage T_1_, the mean survival was 74.86 ± 49.3 months compared to 71.1 ± 47.48 in patients classified as T_2_; patients without lymph node involvement had a mean survival of 74.17 ± 49.0 months compared to 69.55 ± 46.7 months in patients staged as N_1_. The time of recurrence was 38.1 ± 33.9 months in patients classified at tumour stage T_1_ compared with 27.2 ± 22.7 months in patients classified T_2_. In patients without lymph node involvement the time of recurrence was 35.1 ± 30.5 months compared to 25.63 ± 24.6 months in patients with N_1_ disease. A total of 70 patients (21.4%) developed recurrences, 52 patients without lymph node involvement and 18 with lymph node involvement (Table 1). In relation to all patients without lymph node involvement 19.5% developed recurrences (52/266), whereas 26.8% of patients (18/61) with lymph node involvement developed recurrences.

### Univariate analysis

**Figure 1:**
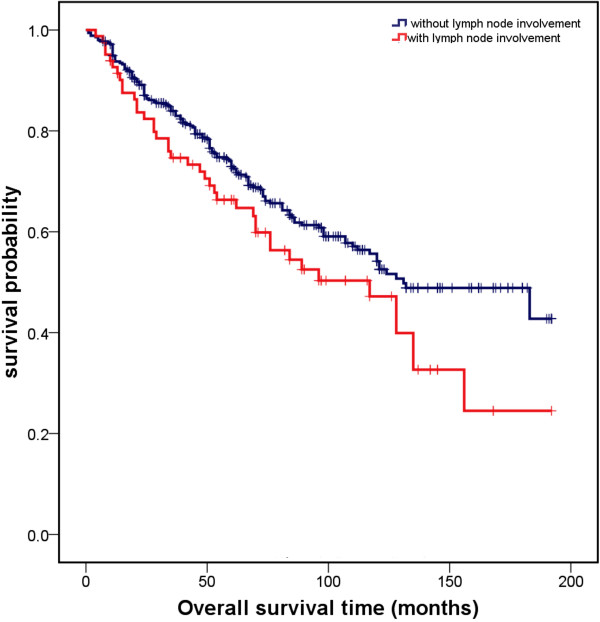
Overall survival curves for lymph node involvement vs. no lymph node metastases significantly influencing survival in univariate analysis (p = 0.049).

**Figure 2:**
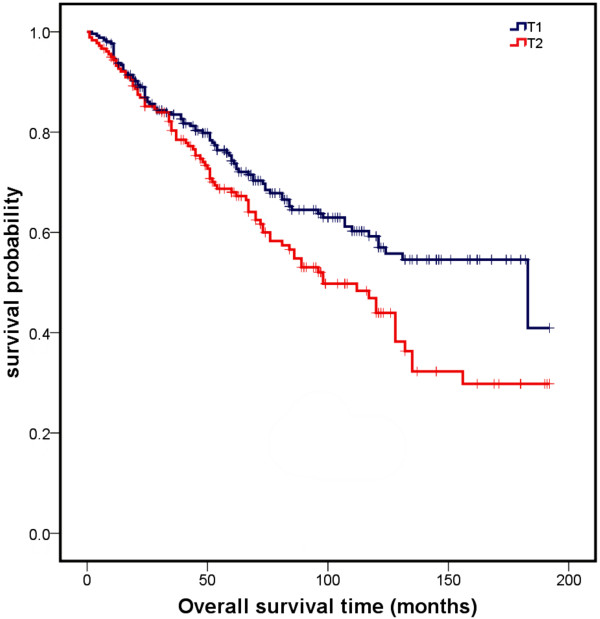
Overall survival curves for the different T stages of tongue carcinomas significantly influencing survival in univariate analysis (p = 0.002).

**Figure 3:**
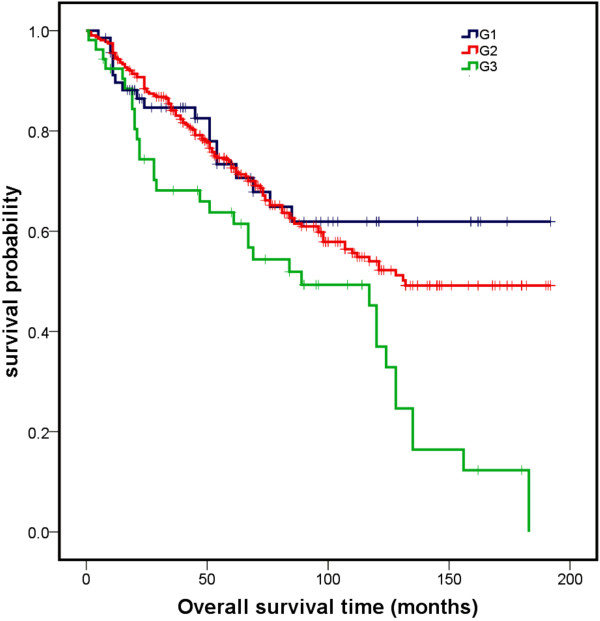
Overall survival curves for the different types of tumour grading significantly influencing survival in univariate analysis.

Variables found to be strongly associated with survival in the univariate analysis included age, tumour and N stage, and grading. The higher the age, tumour and N stage, as well as the grading of the tumour, the more negative was the effect on survival probability. There was no relationship between survival and recurrence or the sex of patient (Table 2). Kaplan-Meier analyses for each variable significantly influencing survival are presented in Figures 1, 2, and 3.

**Table 2:** Univariate Analysis for the factors influencing overall survival (N = 327: 129 death events and 198 censored)

**Variable**	**Hazard ratio**	**95% CI**	**p**
Age	1.03	1.02 to 1.03	< 0.0001*
Tumor stage	1.252	1.16 to 1.35	= 0.01*
N stage	1.60	1.44 to 1.77	= 0.049*
Tumor grade	1.46	1.21 to 1.76	= 0.002*
Sex	1.02	0.83 to 1.25	0.365
Recurrence	1.23	1.00 to 1.52	0.911

Abbreviations: * p < 0.05.

### Multivariate analysis

Cox proportional hazard regression model analysis was conducted controlling for age, tumour stage, nodal stage, tumour grade, and recurrence. Sex was not associated with survival in the univariate model and thus not included in the multivariate analysis. Recurrence was also included into the analysis as it has been reported to be a significant factor associated with survival in the literature.

Age, tumour stage (p = 0.011, 95% CI, 1.09 to 2.0), nodal stage (p = 0.038, 95% CI, 1.02 to 2.07), and recurrence were independently and significantly associated with survival (Table 3). The higher the age, tumour, and nodal stage, the more negative was the effect on survival probability.

**Table 3:** Multivariate Analysis for the factors influencing overall survival (N = 327: 129 death events and 198 censored)

**Variable**	**Hazard ratio**	**95% CI**	**p**
Age	1.04	1.02 to 1.05	< 0.0001*
Sex	1.11	0.73 to 1.71	= 0.622
Tumor stage	1.48	1.09 to 2.0	= 0.011*
N stage	1.68	1.04 to 2.74	= 0.034*
Recurrence	1.79	1.09 to 2.98	= 0.022*
Tumor grade	1.54	1.16 to 2.05	= 0.003*

Abbreviations: * p < 0.05.

## Discussion

In this study we reported the incidence of clinical occult metastases in patients with T_1_ and T_2_ tongue squamous cell carcinomas. Our outcomes have been presented followed by conclusions based on our experience.

Our overall survival rates of our population of patients are comparable with those from other studies and much better than the frequently quoted crude overall survival from oral cancer of 56% [7-9]. Therefore our results may be extrapolated to patients from other countries with similar staging. Management of the N_0_ neck in head and neck squamous cell carcinoma is an important issue for the head and neck surgeon. In patients with head and neck cancer, the presence of lymph nodes metastases is the most important prognostic factor [10]. The management of patients with clinically negative nodes (N_0_) with early tongue cancer is controversial. There are available papers in the literature but the results of these have not provided a satisfactory answer to the controversy [11-14]. There is a single randomised controlled trial attempting to provide an evidence base for guidance on the definition of a threshold of risk over which a neck dissection is indicated. It is widely believed that tumour thickness will be the most useful single determinant for this decision. Weiss et al. 1994 concluded that N0 necks should be treated electively when the occult metastatic rate is more than 20% [15].

In 61/327 patients lymph node involvement was demonstrated. Studies have shown that a significant number of patients with early oral cavity cancers who are observed will develop neck recurrence and many of these recurrences will be of advanced stage with poor prognostic factors such as extra-capsular spread (ECS) [13]. The salvage rates in these patients with recurrences were found to be only 30% [12]. However we must bear in mind that there are no available randomised controlled trials comparing prophylactic treatment of the N_0_ neck with observation and therapeutic neck dissection on detection of recurrence. There is a body of evidence from retrospective studies suggesting that in patients who do not have prophylactic therapy of the clinically N_0_ neck there is often a low salvage rate on disease recurrence [16].

The diagnosis and treatment of oral cancer are negative predictors of health-related quality of life. In the oral cavity both, a “wait and see” policy or an elective selective neck dissection may be successful approaches tailored to specific patients. The reality is that some patients with oral cancer and N_0_ neck do not have occult metastases and would not benefit from an elective neck dissection, some may have micrometastases but there is no certainty that these will necessarily progress. There are a group of patients with occult metastases which do present later after primary treatment and have a poorer outcome than would have been anticipated from their original disease [9,12]. Current data has been unable to provide recommendations based on sound evidence. Especially in the clinical negative neck, shown in this study, surprisingly, there is a high rate of occult metastases within the lymph nodes of 18.65% of patients. In the present study a large amount of patients were included and even higher lymph node metastases rates have been reported up to 42% in the past [12,13].

## Conclusion

Even though staging of patients is still increasing by better CT and MRI scanning there is a lack of evidence that patients with negative lymph node staging would benefit from neck preservation. When we assess the literature we must recognize that the poor salvage rates recorded were based on work that was completed in the 80’s. Overall as surgeons we may observe the neck more frequently and this may be due to advances in the examination of nodal status and potentially the higher detection rates of patients with early lymph node metastases. Nevertheless, as stated in the guidelines of oral cavity cancer neck dissections should be performed as part of the concept to complete the oncological therapy of this complex tumour entity including its biological behaviour [17].

## Competing interests

The authors declare that they have no competing interests.

## Authors’ contributions

TM: conception and design; acquisition of data; drafting and revising the manuscript; DAM: conception and design; acquisition of data; drafting and revising the manuscript. SW: analysis and interpretation of data; drafting and revising the manuscript. LMR: analysis and interpretation of data; drafting and revising the manuscript; KDW: analysis and interpretation of data; drafting and revising the manuscript; AK: conception and design; acquisition of data; drafting and revising the manuscript; All authors read and approved the final manuscript and agree to be accountable for all aspects of the work in ensuring that questions related to the accuracy or integrity of any part of the work are appropriately investigated and resolved.

## Pre-publication history

The pre-publication history for this paper can be accessed here:

http://www.biomedcentral.com/1471-2407/14/346/prepub

## References

[B1] Shah JP Gil Z Current concepts in management of oral cancer–surgery Oral Oncol 2009 45 4–5 394 401 18674952 10.1016/j.oraloncology.2008.05.017 PMC4130348

[B2] Shingaki S Takada M Sasai K Bibi R Kobayashi T Nomura T Saito C Impact of lymph node metastasis on the pattern of failure and survival in oral carcinomas Am J Surg 2003 185 3 278 284 10.1016/S0002-9610(02)01378-8 12620571

[B3] Mücke T Koschinski J Wagenpfeil S Wolff KD Kanatas A Mitchell DA Deppe H Kesting MR Functional outcome after different oncological interventions in head and neck cancer patients J Cancer Res Clin Oncol 2012 138 3 371 376 10.1007/s00432-011-1106-x 22143103 PMC11824191

[B4] Bar Ad V Chalian A Management of clinically negative neck for the patients with head and neck squamous cell carcinomas in the modern era Oral Oncol 2008 44 9 817 822 10.1016/j.oraloncology.2007.12.003 18328776

[B5] Elsheikh MN Rinaldo A Ferlito A Fagan JJ Suarez C Lowry J Paleri V Khafif A Olofsson J Elective supraomohyoid neck dissection for oral cavity squamous cell carcinoma: is dissection of sublevel IIB necessary? Oral Oncol 2008 44 3 216 219 10.1016/j.oraloncology.2007.06.006 17826302

[B6] Mücke T Wolff KD Wagenpfeil S Mitchell DA Hölzle F Immediate microsurgical reconstruction after tumor ablation predicts survival among patients with head and neck carcinoma Ann Surg Oncol 2010 17 1 287 295 10.1245/s10434-009-0758-0 19841982

[B7] Brown JS Magennis P Rogers SN Cawood JI Howell R Vaughan ED Trends in head and neck microvascular reconstructive surgery in Liverpool (1992–2001) Br J Oral Maxillofac Surg 2006 44 5 364 370 10.1016/j.bjoms.2005.07.018 16169640

[B8] Brown JS Rogers SN Lowe D A comparison of tongue and soft palate squamous cell carcinoma treated by primary surgery in terms of survival and quality of life outcomes Int J Oral Maxillofac Surg 2006 35 3 208 214 10.1016/j.ijom.2005.09.005 16343850

[B9] Zhang T Lubek JE Salama A Dyalram D Liu X Ord RA Treatment of cT1N0M0 tongue cancer: outcome and prognostic parameters J Oral Maxillofac Surg 2013 72 2 406 414 24045188 10.1016/j.joms.2013.05.028

[B10] Woolgar JA Histopathological prognosticators in oral and oropharyngeal squamous cell carcinoma Oral Oncol 2006 42 3 229 239 10.1016/j.oraloncology.2005.05.008 16150633

[B11] Fakih AR Rao RS Patel AR Prophylactic neck dissection in squamous cell carcinoma of oral tongue: a prospective randomized study Semin Surg Oncol 1989 5 5 327 330 10.1002/ssu.2980050507 2682926

[B12] Ho CM Lam KH Wei WI Lau SK Lam LK Occult lymph node metastasis in small oral tongue cancers Head Neck 1992 14 5 359 363 10.1002/hed.2880140504 1399568

[B13] Kligerman J Lima RA Soares JR Prado L Dias FL Freitas EQ Olivatto LO Supraomohyoid neck dissection in the treatment of T1/T2 squamous cell carcinoma of oral cavity Am J Surg 1994 168 5 391 394 10.1016/S0002-9610(05)80082-0 7977957

[B14] Yuen AP Ho CM Chow TL Tang LC Cheung WY Ng RW Wei WI Kong CK Book KS Yuen WC Trendell-Smith NJ Chan YW Wong BY Li GK Ho AC Womg SY Yao TJ Prospective randomized study of selective neck dissection versus observation for N0 neck of early tongue carcinoma Head Neck 2009 31 6 765 772 10.1002/hed.21033 19408291

[B15] Weiss MH Harrison LB Isaacs RS Use of decision analysis in planning a management strategy for the stage N0 neck Arch Otolaryngol Head Neck Surg 1994 120 7 699 702 10.1001/archotol.1994.01880310005001 8018319

[B16] Leon X Quer M Orus C Sancho FJ Bague S Burgues J Selective dissection of levels II-III with intraoperative control of the upper and middle jugular nodes: a therapeutic option for the N0 neck Head Neck 2001 23 6 441 446 10.1002/hed.1148 11360304

[B17] Wolff KD Follmann M Nast A The diagnosis and treatment of oral cavity cancer Dtsch Arztebl Int 2012 109 48 829 835 23248713 10.3238/arztebl.2012.0829 PMC3523261

